# Ceftriaxone-Resistant Salmonella enterica Serotype Typhimurium Sequence Type 313 from Kenyan Patients Is Associated with the *bla*_CTX-M-15_ Gene on a Novel IncHI2 Plasmid

**DOI:** 10.1128/AAC.00078-15

**Published:** 2015-05-14

**Authors:** Samuel Kariuki, Chinyere Okoro, John Kiiru, Samuel Njoroge, Geoffrey Omuse, Gemma Langridge, Robert A. Kingsley, Gordon Dougan, Gunturu Revathi

**Affiliations:** a Centre for Microbiology Research, Kenya Medical Research Institute, Nairobi, Kenya; b Aga Khan University Hospital, Nairobi, Kenya; c The Wellcome Trust Sanger Institute, Wellcome Trust Genome Campus, Hinxton, Cambridge, United Kingdom

## Abstract

Multidrug-resistant bacteria pose a major challenge to the clinical management of infections in resource-poor settings. Although nontyphoidal Salmonella (NTS) bacteria cause predominantly enteric self-limiting illness in developed countries, NTS is responsible for a huge burden of life-threatening bloodstream infections in sub-Saharan Africa. Here, we characterized nine *S*. Typhimurium isolates from an outbreak involving patients who initially failed to respond to ceftriaxone treatment at a referral hospital in Kenya. These Salmonella enterica serotype Typhimurium isolates were resistant to ampicillin, chloramphenicol, cefuroxime, ceftriaxone, aztreonam, cefepime, sulfamethoxazole-trimethoprim, and cefpodoxime. Resistance to β-lactams, including to ceftriaxone, was associated with carriage of a combination of *bla*_CTX-M-15_, *bla*_OXA-1_, and *bla*_TEM-1_ genes. The genes encoding resistance to heavy-metal ions were borne on the novel IncHI2 plasmid pKST313, which also carried a pair of class 1 integrons. All nine isolates formed a single clade within *S*. Typhimurium ST313, the major clone of an ongoing invasive NTS epidemic in the region. This emerging ceftriaxone-resistant clone may pose a major challenge in the management of invasive NTS in sub-Saharan Africa.

## INTRODUCTION

It is estimated that nontyphoidal Salmonella (NTS) causes ∼100 million cases of gastroenteritis every year, of which ∼155,000 are fatal (1). Although NTS predominantly causes localized gut infections, invasive NTS (iNTS) infections may occur when the bacteria spread beyond the mucosa to reach normally sterile sites, such as the bloodstream, bones, joints, and the brain (2). A significant burden of iNTS cases are found in sub-Saharan Africa (3, 4), caused mainly by Salmonella enterica serotypes Typhimurium and Enteritidis (5, 6). Infections with these serotypes are associated with poor outcomes, including a high rate of mortality, especially among children less than 5 years of age and in HIV-infected adults who have low CD4 T-lymphocyte counts (7, 8). Due to an increasing prevalence of resistance to commonly available antibiotics, extended-spectrum cephalosporins and fluoroquinolones now are recommended for the management of iNTS (9). However, these alternative antimicrobials are less widely available and more expensive to use in resource-limited settings. Although resistance to first-line antimicrobials, such as ampicillin, chloramphenicol, and cotrimoxazole, is common among NTS from Kenya and elsewhere in sub-Saharan Africa, resistance to ceftriaxone and fluoroquinolones has been reported rarely.

Whole-genome sequence analysis of *S*. Typhimurium, isolated in a number of countries in sub-Saharan Africa, has identified a novel multilocus sequence type (MLST), ST313, which is responsible for an ongoing epidemic in the region (10). These ST313 *S*. Typhimurium strains fall into two related ST313 clades and encode multidrug resistance (MDR), in part on integrons associated with the invasion-associated plasmid pSLT. Here, we report the isolation and genomic characterization of ST313 *S*. Typhimurium isolates from patients seeking treatment in a tertiary-care and teaching hospital in Nairobi, Kenya, that exhibited resistance to ceftriaxone with or without combined resistance to fluoroquinolones.

## MATERIALS AND METHODS

### Patients and NTS strains.

Adults and children reporting with fever to the Aga Khan University Hospital in Nairobi, Kenya, and referred for blood culture investigations between April 2009 and December 2011 were included in this study (Table 1). These patients were admitted with severe sepsis and failed treatment with routinely used antimicrobials, including ceftriaxone. Meropenem or imipenem was used to treat the patients by following Sanford guidelines (2012) on antimicrobial therapy (30).

**TABLE 1:** Susceptibility profiles of *S*. Typhimurium isolates recovered from different specimen types and patient categories

Isolate ID	Source specimen	Patient age	Patient category	Date of isolation	Susceptibility profile^*a*^						ESBL test
					NAL	CIP	CRO	AMP	CHL	SXT	
1891	Stool	43 yr	Inpatient	April 2010	S	S	R	R	R	R	Positive
8892	Urine	1 mo	Inpatient	May 2010	S	S	R	R	R	R	Positive
8943	Stool	2 yr	Inpatient	February 2012	S	S	R	R	R	R	Positive
8947	Stool	33 yr	Outpatient	October 2010	S	S	R	R	R	R	Positive
8942	Blood	4 yr	Inpatient	June 2009	S	S	R	R	R	R	Positive
8945	Stool	Adult (age not known)	Outpatient	July 2009	S	S	R	R	R	R	Positive
8946	Stool	Child (age not known)	Outpatient	December 2011	S	S	R	R	R	R	Positive
8944	Blood	Child (age not known)	Inpatient	December 2011	S	S	R	R	R	R	Positive
8948	Stool	Child (age not known)	Outpatient	December 2011	S	S	R	R	R	R	Positive

a Susceptibility profiles of isolates analyzed in this study. NAL, nalidixic acid; CIP, ciprofloxacin; CRO, ceftriaxone; AMP, ampicillin; CHL, chloramphenicol; SXT, sulfamethoxazole-trimethoprim; ESBL test, ESBL status confirmed using the double disc synergy method. R, resistant; S, susceptible.

Blood specimens from children and adults were inoculated into blood culture media and incubated in a Bactec 9050 culture system (Becton, Dickinson and Company). All positive blood cultures were subcultured onto sheep blood agar, Salmonella-Shigella agar (SSA), and MacConkey agar plates (Oxoid, Basingstoke, United Kingdom). In addition, fecal samples from patients who had diarrhea initially were enriched in selenite F broth and subcultured on MacConkey agar and SSA (Oxoid). Bacterial isolates were identified by biochemical tests using API 20E strips (bioMérieux, Basingstoke, United Kingdom) and serotyped using agglutinating antisera (Murex Diagnostics, Dartford, United Kingdom). *S*. Typhimurium isolates were stored at −70°C on Protect beads (Technical Service Consultants Ltd., Heywood, United Kingdom).

### Antimicrobial susceptibility testing.

NTS isolates were tested for susceptibility to antimicrobials by a controlled disk diffusion technique on diagnostic sensitivity testing (DST) agar (Oxoid) plates. The antibiotic discs (all from Oxoid) contained ampicillin (10 μg), tetracycline (30 μg), cotrimoxazole (1:25 μg), chloramphenicol (30 μg), gentamicin (10 μg), coamoxyclav (20:10 μg), ciprofloxacin (10 μg), ceftriaxone (30 μg), and nalidixic acid (10 μg). Escherichia coli ATCC 25922 was included to control for disc potency and quality of the culture media. Susceptibility tests were interpreted using the Clinical and Laboratory Standards Institute (CLSI) guidelines (11). In addition, MICs were performed with an automated bacteriology analyzer, Vitek Compact 2 (bioMérieux), using Gram-negative card GN26, which had the following antimicrobials that were clinically relevant to Salmonella spp.: ampicillin, ceftriaxone, ciprofloxacin, cefepime, trimethoprim-sulfamethoxazole, and meropenem. Cefpodoxime, cefoxitin, aztreonam, and ceftazidime are not clinically relevant to management of iNTS infections but were used as extended-spectrum beta lactamase (ESBL) markers. Double disk synergy tests for ESBL confirmation were performed using previously described methods (12). Current CLSI updates on interpretation of ciprofloxacin susceptibility cutoffs on Salmonella spp. were utilized when reporting susceptibility results of blood culture isolates to clinicians for management of patients.

### Genomic DNA preparation.

Bacteria for genomic analysis first were grown on Luria-Bertani (LB) medium (Oxoid) by inoculating a single isolated colony into broth, followed by incubation overnight at 37°C. The bacterial growth was pelleted by centrifugation, and whole-genome DNA was extracted using the Wizard genomic DNA kit (Promega, Southampton, United Kingdom). Aliquots of 20 to 50 ng/μl of DNA from each isolate were submitted for whole-genome sequencing.

### Library preparation and DNA sequencing.

Multiplex libraries with 108-bp reads and a mean insert size of 272 bp were prepared as previously described (13). The cluster formation, primer hybridization, and sequencing reactions were performed using the Illumina Hiseq sequencer (LGC, Middlesex, United Kingdom). The resulting short-read sequences were deposited in the European Nucleotide Archive; accession numbers for the whole genome are listed below. An average of 91.7% of each strain was mapped using SMALT with a mean depth of 118.6-fold coverage in mapped regions across all isolates, as previously described (14).

### Phylogenetic analysis.

Maximum-likelihood phylogenetic trees were constructed using RAxML v7.0.4 (14), with an alignment of all the concatenated variant sites from the nonrepetitive and nonrecombinant genome included in the analyses as previously described (14). The maximum-likelihood ratios then were calculated using the general time-reversible model with a gamma correction for site variation as the nucleotide substitution model. The likelihood test ratios were determined as previously described (10). The support for nodes on the trees was checked using 100 random bootstrap replicates. Resulting phylogenetic trees were visualized using the FigTree package v1.4.0 (http://tree.bio.ed.ac.uk/software/figtree/).

### PacBio sequencing and assembly.

Sequencing was performed using the PacBio template preparation kit (PacBio, Menlo Park, CA, USA) by following a modified version of the 20-kb template preparation using the BluePippin size selection system protocol to prepare size-selected libraries from 5 μg of sheared DNA. Further sequencing was performed using the magnetic bead collection protocol, a 20,000-bp insert size, stage start, and 180-min movies to set up the run protocol in RS Remote. In order to capture smaller-sized plasmids, the 5-kb template preparation protocol was applied. *De novo* assembly was performed using the standard SMRT Analysis (v. 2.0.2) pipeline.

### Plasmid genome analyses.

The plasmid map was annotated using DNAPlotter 10.2. The identity of genes of interest was confirmed using NCBI tools, including BLAST. Mutations were detected using the protein sequence alignment CLUSTALW program (15). The identification of beta-lactamase gene mutations at the amino acid level was determined by comparing the amino acid sequence of the sequenced enzyme to that of the wild-type enzyme published at http://www.lahey.org/studies/.

### Study ethical approval.

Ethical approval for the study was obtained from the KEMRI Ethical Review Committee (SSC no. 2027).

### Nucleotide sequence accession numbers.

Accession numbers for the whole-genome short-read sequences for each of the isolates analyzed are the following: 8947, ERR387652; 8944, ERR387655; 8942, ERR387656; 8948, ERR387657; 8943, ERR387720; 8945, ERR387721; 8946, ERR387722; 5580, ERR034067; 5597, ERR034068; 5575, ERR034078; 5582, ERR034079; 5576, ERR034080; 5912, ERR034081; 5647, ERR034082; 5634, ERR034083; 5632, ERR034084; 5581, ERR034086; 5578, ERR034087; 5577, ERR034088; A130, ERR023781; 8891, ERR044726; 8892, ERR044727; D2358, FN424405; and SL1344, FQ312003. The nucleotide sequence data for the pSBLT and pKST313 plasmids were submitted to the National Center for Biotechnology Information Data Libraries (GenBank), and the sequences have been given accession numbers LN794247 and LN794248, respectively.

## RESULTS

### Antimicrobial susceptibility and ESBL testing.

The antimicrobial resistance profiles of the nine NTS isolates were identical, regardless of the source. These isolates also were positive for ESBL production by double disc synergy and MIC testing but were susceptible to ciprofloxacin and meropenem. In addition, all isolates also were resistant to ampicillin, chloramphenicol, cefuroxime, ceftriaxone, cefotaxime, aztreonam, cefepime, and sulfamethoxazole-trimethoprim (Table 2).

**TABLE 2:** MICs of isolates analyzed

Isolate ID	MIC^*a*^ (μg/ml)									
	CIP	AMP	SXT	CRO	CTX	MEM	FOX	CPD	FEP	ATM
1891	0. 006	>32	>320	>256	>64	<0.25	2	>8	>64	>64
8892	0.012	>32	>320	>256	>64	<0.25	2	>8	>64	.>64
8943	<0.25	>32	>320	>64	>64	<0.25	8	>8	>64	>64
8947	<0.25	>32	>320	>64	>64	<0.25	8	>8	>64	>64
8942	<0.25	>32	>320	>64	>64	<0.25	8	>8	>64	>64
8945	<0.25	>32	>320	>64	>64	<0.25	8	>8	>64	>64
8946	<0.25	>32	>320	>64	>64	<0.25	8	>8	>64	>64
8944	<0.25	>32	>320	>64	>64	<0.25	8	>8	>64	>64
8948	<0.25	>32	>320	>64	>64	<0.25	8	>8	>64	>64

a MICs against selected antimicrobials. CIP, ciprofloxacin; AMP, ampicillin; SXT, sulfamethoxazole-trimethoprim; CRO, ceftriaxone; CTX, cefotaxime; MEM, meropenem; FOX, cefoxitin; CPD, cefpodoxime; FEP, cefepime; ATM, aztreonam.

### The ESBL-producing NTS isolates are ST313.

**FIG 1:**
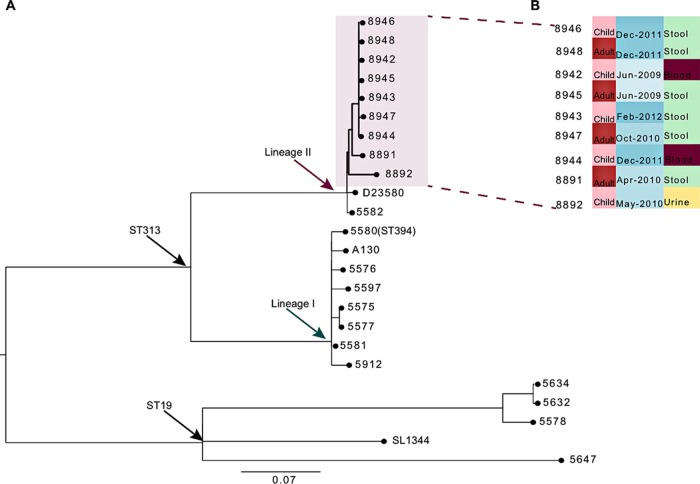
Phylogenetic relationships between isolates. Unrooted maximum-likelihood tree showing the relationship between isolates (pink polygon) and other Kenyan ST313 organisms isolated between 2003 and 2005 (10). D23580 (lineage II), A130 (lineage I), and the reference isolate SL1344 (ST19) are indicated on the tree. Scale bars represent the number of substitutions that have occurred on each branch. The inset in panel B represents associated metadata of the isolate arranged to reflect phylogenetic positioning.

Whole-genome sequence analyses of the nine isolates confirmed they belonged to the same ST313 clade that has been isolated frequently in the sub-Saharan Africa region (10). Phylogenetic analyses based on whole-genome single-nucleotide polymorphisms (SNPs) assigned as previously reported for ST313 (10) showed that these ESBL-producing strains fell into clade II and were highly related to the reference ST313 invasive isolate D23580 from Malawi, with a mean SNP difference of 39 (range, 31 to 70). A mean SNP difference between each pair of only 21 (range, 0 to 70) meant that the nine isolates were even more closely related to each other than to D23580 or any other representative *S*. Typhimurium lineages, including the closest Kenyan strains described from previous outbreaks (Fig. 1). Thus, these isolates potentially represent a clone circulating within the study population. In addition, our pairwise comparison also showed that seven of these isolates, 8942, 8944, 8947, 8943, 8945, 8946, and 8948, harbor minimal genome differences, represented by only a 1- or 2-SNP difference. The other two isolates, 8891 and 8892, occupy a different branch of the tree and were separated from the others by an average of 48 SNPs (range, 27 to 69) (Table 2). Overall, the low chromosomal SNP differences indicate a clonal outbreak within the study population, especially given the clustering away from the historical Kenyan ST313 isolates on the tree (Fig. 1).

**FIG 2:**
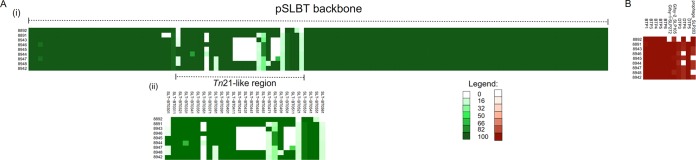
Distributions of known virulence-associated determinants in isolates. (Ai) Spaces/gaps represent genes missing from some of the isolates. (Aii) Presence of the Tn*21*-like region in pSLBT plasmids in the isolates. Numbers at the end of gene names represent accession codes for various genes associated with Tn*21*. The genes found in the isolates are presented as heatmaps against various genes present in Tn*21*. Color gradients shows full coverage (with the darkest shade representing 100% match) to no coverage (lightest shade representing 0% match) of the genes present in the Tn*21*-like region in pSLBT plasmids of the study isolates against those found on a typical Tn*21* region.

**FIG 3:**
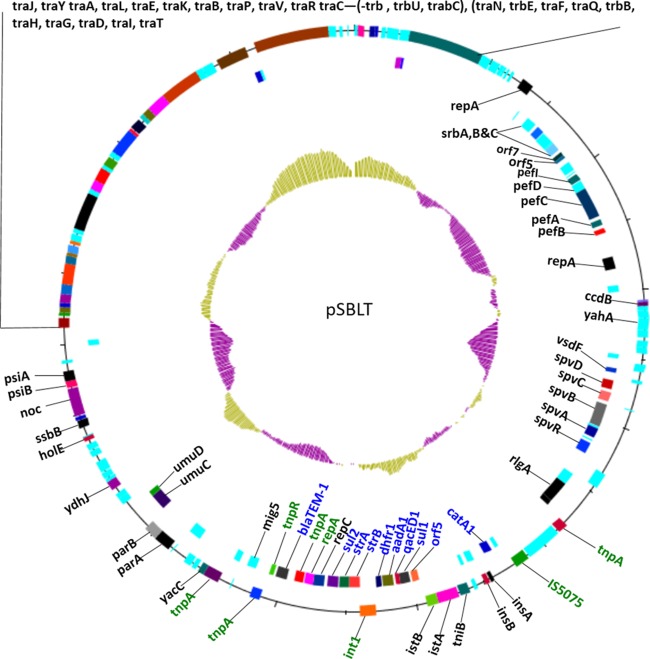
Genetic map of the pSLBT plasmid. Only genes with predicted functions are labeled. Genes involved in resistance to antimicrobials are labeled in blue. Genes related to integrons, IS, and transposons are labeled in green. The first outer ring represents the forward open reading frame, the second inner ring shows the reverse open reading frames, and the inner most circle is the % GC plot (yellow, above average; purple, below average).

We next analyzed these isolates for characteristic genomic markers that have been described for the ST313 lineages, including plasmids, phage repertoire, and antibiotic resistance genes. A pSLBT IncFII plasmid similar to the multidrug-resistant plasmid found in D23580 (6) was identified in all isolates (Fig. 2A, i and ii). These isolates also contained a conserved repertoire of phages similar to that of D23580 (Fig. 2B). The pSLBT plasmid carried resistance determinants for aminoglycosides (*aadA1*), streptomycin (*strA* and *strB*), β-lactams (*bla*_TEM-1_), chloramphenicol (*catA1*), trimethoprim (*dhfr1*), and sulfonamides (*sul1* and *sul2*). These resistance genes were located in a region containing a degenerate Tn*21* transposon (Fig. 3).

### Carriage of IncHI2 plasmids among the ST313 isolates.

**FIG 4:**
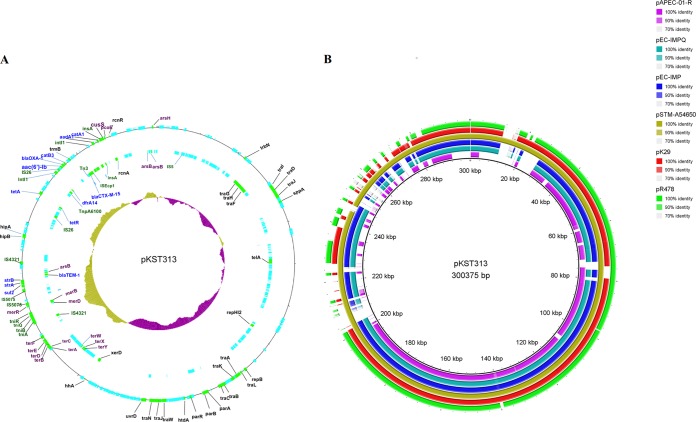
(A) Genetic map of the pKST313 plasmid. Genes involved in resistance to antimicrobials are labeled in blue, while those related to (heavy) metal resistance are labeled in purple. Genes related to integrons, IS, and transposons are labeled in green. The outer ring represents the forward open reading frames, the second ring represents the reverse open reading frames, and the innermost circle is the % GC plot (yellow, above average; purple, below average). (B) Pairwise comparisons of other IncHI2 plasmids. The innermost black ring 1 represents the reference sequence of pKST313. Six annotated plasmids with their respective accession numbers used for the comparison are the following: ring 2, pAPEC-01-R (DQ517526); ring 3, pEC-IMPQ (EU855787); ring 4, pEC-IMP (EU855787); ring 5, pSTM-A54650 (LK056646); ring 6, pK29 (EF382672); and ring 7 (the outermost ring), pR478 (BX664015). Gaps represent diversity from the reference sequence (pKST313).

In addition to the pSLBT IncFII plasmid, the nine ESBL-producing ST313 isolates contained an ∼300-kb IncHI2 plasmid, which we designate pKST313 (for plasmid from the ST313 Kenyan strain). pKST313 encodes two class 1 integrons, various transposons, multiple insertion sequence (IS) elements, and multiple resistance cassettes (Fig. 4A and B). One region of the plasmid encodes resistance to heavy-metals ions, including mercury (*mer* and *tni* genes), tellurite (*ter* genes), arsenic (*ars* genes), and copper (*cusS* and *pcoE* genes). This region also contained tellurite utilization genes (*tel* genes). A second region harbors genes conferring resistance to antimicrobials, including aminoglycosides (*aadA1*), streptomycin (*strA* and *strB*), β-lactams (*bla*_TEM-1_), chloramphenicol (*catA1*), trimethoprim (*dhfr1*), and sulfonamides (*sul1* and *sul2*), the majority of which are within a class 1 integron. The third region (between *trbN* and *traN*) (Fig. 3) contained genes responsible for plasmid replication, pilus formations, and conjugation. The gene encoding DNA repair protein UvrD also was found adjacent to this region. This plasmid also contained multiple ISs that include IS*Ecp1*, associated with *bla*_CTXM-15_, the integron-associated IS*26*, IS*4321* and IS*5075*, located near the mercury resistance region, IS*6100*, and IS*5*.

### Redundancy in antimicrobial resistance genes/cassettes.

These *S*. Typhimurium ST313 isolates carried multiple genes/cassettes conferring resistance to the same class of antimicrobials. Such genes/cassettes included two copies of *strA* and *strB* (one pair in the pSLBT plasmid and the other in the pKST313 plasmid) conferring resistance to aminoglycosides. The *catA1* gene (in the pSLBT plasmid) and both *catA1* and *catB3* cassettes (in the pKST313 plasmid) encode resistance to chloramphenicol. The isolates also carried a combination of *aac*(*6*′)-Ib and a single copy of *aadA1* in each plasmid, all encoding resistance to aminoglycosides. All of the isolates had multiple copies of β-lactamases that included *bla*_TEM-1_ in both plasmids, *bla*_OXA-1_ (that is poorly inhibited by clavulanic acid), and *bla*_CTX-M-15_ (in pKST313). The *dfrA1* cassette conferring resistance to trimethoprim was found in pSLBT, while pKST313 carried another trimethoprim resistance gene, *dfrA14*. The most redundant resistance sets of genes were those conferring resistance to sulfonamides, with pKST313 carrying both the *sul1* and *sul2* gene, while a second copy of *sul2* was located on the pKST313 plasmid.

## DISCUSSION

Although ceftriaxone-resistant *S*. Typhimurium strains have been reported previously in other countries in Europe (16), Asia (17, 18), and the United States (19, 20), few data are available on this phenotype in sub-Saharan Africa. *S*. Typhimurium ST313 is the dominant pathovariant of invasive NTS disease in immunocompromised adults and children in sub-Saharan Africa, and it is noteworthy that the acquisition of this new plasmid has occurred within this clonal population. We describe clonally related MDR *S*. Typhimurium ST313 isolates from adults and children who reported to a referral hospital with fever, with or without diarrhea, and initially were treated with ceftriaxone and failed to respond. The spectrum of resistance in these isolates extends beyond the cephalosporins to include tetracyclines, chloramphenicol, and aminoglycosides. Although high MIC values for cotrimoxazole, cefotaxime, ceftriaxone, and aztreonam were recorded (Table 2), these isolates remained susceptible to carbapenems and fluoroquinolones. Locally, ceftriaxone has been the drug of choice for use against iNTS that are resistant to commonly available antimicrobials. Interestingly, none of these isolates was found to contain a cephamycin resistance gene, such as the *bla*_CMY_ gene. These isolates also were resistant to co-amoxiclav but did not carry any known inhibitor resistance genes, such as those that encode inhibitor-resistant TEMs (IRTs). A possible explanation for these unexpected observations is that these strains carry multiple copies of *bla* genes, namely, *bla*_OXA-1_, *bla*_TEM-1_, and *bla*_CTX-M-15_ genes. The *bla*_OXA-1_ gene has been reported to confer resistance to inhibitor β-lactams; therefore, the spread of this gene may limit the utility of such agents (21). The *bla*_CTX-M-15_ gene detected in these isolates encodes the most robust CTX-M-type ESBLs described to date and has a wider hydrolytic capability against most cephalosporins, including ceftazidime, than other CTX-Ms. The *bla*_CTX-M-15_ gene was physically linked to the IS*Ecp1* element that has been implicated in the expression and transposition of this gene and has been linked to global dissemination in E. coli.

The NTS described in this study carried two plasmids that encode resistance to multiple antimicrobials. The pSLBT plasmid also harbored multiple virulence genes and integron-borne resistance determinants as previously described (6). However, the majority of resistance genes in these isolates were borne on pKST313, a significant portion of which is dedicated to antibiotic resistance and resistance to heavy-metal ions. This plasmid could play a critical role in the persistence of NTS and other bacteria in environments heavily contaminated with detergents, heavy metals, and other antimicrobials (22).

We found the Tn*3-bla*_TEM-1_-*strB-strA-Sul2* gene motif, which has been described as a promiscuous sequence that has the potential to spread to different types of plasmids. As reported in other studies, this gene motif was closely associated with IS*26* and IS*4321*. The IS*26*-associated *bla*_TEM-1_-*strB-strA-sul2* sequences have been reported on other plasmids, such as pHCM1 (23) and pAKU1 (24). Similar sequences also have been reported in other IncF plasmids, such as the 120-kb IncF plasmid isolated in Germany in 2005 (designated pRSB107) (25) and the IncF-like plasmid pU302L of *S*. Typhimurium G8430 (26).

Previous studies have indicated that bacteria bearing a combination of antimicrobial and heavy-metal resistance genes are released from poorly treated wastewater treatment plants (27, 28). It is important to note that pKST313 shares over 80% of resistance genes and (mobile) genetic elements with an IncHI2 (pK29) plasmid found in some clinical Klebsiella pneumoniae strains, while the metal resistance region is similar to determinants found on plasmids in E. coli, Pseudomonas spp., and the soil bacteria Rhizobium spp. (29). These findings suggest that the novel plasmid originated from the environment and is a hybrid of different plasmids, IS elements, and transposons brought together by recombination. Comparative analysis indicates that IS*26* detected in multiple copies can act as a recombination junction between plasmid ancestors that can lead to the emergence of new phenotypes.

In conclusion, we observed in Kenya the emergence of ceftriaxone-resistant *S*. Typhimurium ST313 that has been associated with an epidemic of invasive bacterial disease. This resistance and MDR phenotype are borne in part on the pKST313 plasmid, which contains integrative elements with the potential to rapidly disseminate. These data highlight the threat of an epidemic shaped by this new MDR phenotype.
